# Digital PCR as a tool to measure HIV persistence

**DOI:** 10.1186/s12977-018-0399-0

**Published:** 2018-01-30

**Authors:** Sofie Rutsaert, Kobus Bosman, Wim Trypsteen, Monique Nijhuis, Linos Vandekerckhove

**Affiliations:** 1 HIV Cure Research Center, Department of Internal Medicine, Ghent University, Ghent, Belgium; 2 Department of Medical Microbiology, Virology, UMC Utrecht, Utrecht, The Netherlands

**Keywords:** Digital PCR, HIV, ddPCR

## Abstract

Although antiretroviral therapy is able to suppress HIV replication in infected patients, the virus persists and rebounds when treatment is stopped. In order to find a cure that can eradicate the latent reservoir, one must be able to quantify the persisting virus. Traditionally, HIV persistence studies have used real-time PCR (qPCR) to measure the viral reservoir represented by HIV DNA and RNA. Most recently, digital PCR is gaining popularity as a novel approach to nucleic acid quantification as it allows for absolute target quantification. Various commercial digital PCR platforms are nowadays available that implement the principle of digital PCR, of which Bio-Rad’s QX200 ddPCR is currently the most used platform in HIV research. Quantification of HIV by digital PCR is proving to be a valuable improvement over qPCR as it is argued to have a higher robustness to mismatches between the primers-probe set and heterogeneous HIV, and forfeits the need for a standard curve, both of which are known to complicate reliable quantification. However, currently available digital PCR platforms occasionally struggle with unexplained false-positive partitions, and reliable segregation between positive and negative droplets remains disputed. Future developments and advancements of the digital PCR technology are promising to aid in the accurate quantification and characterization of the persistent HIV reservoir.

## Background

**Fig. 1:**
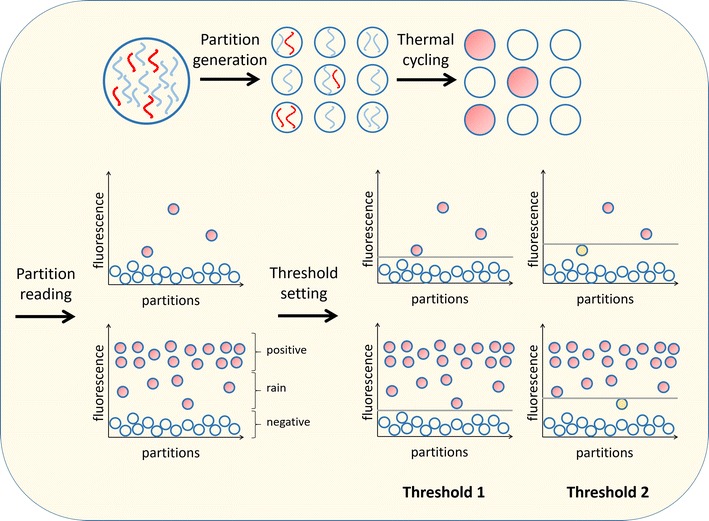
Digital PCR. In digital PCR the sample is divided in multiple partitions. After PCR amplification, partitions containing the target produce a signal and are assigned positive. Discriminating between positive and negative partitions remains challenging and threshold setting can influence quantification, especially in low target settings

During antiretroviral therapy (ART), HIV can persist for decades in latently infected CD4 + T cells as proviral DNA integrated in the human genome. If ART is interrupted, the proviral reservoir fuels rebound viremia and is therefore considered a major obstacle to HIV cure [1]. HIV cure efforts aim to reduce the size and replication-competence of the reservoir by evaluating the success of HIV cure interventions, which is represented by an effect on the level of proviral DNA and/or cell-associated viral RNA. The standard tool to quantify HIV DNA and cell-associated viral RNA has been real-time PCR (qPCR). However, digital PCR has become a promising quantification strategy that combines absolute quantification with high sensitivity [2]. Digital PCR is based on the concept of limiting dilution where target molecules are randomly divided among a multitude of partitions. After PCR amplification, partitions that contain a target molecule accumulate fluorescence whereas partitions without target remain low in fluorescence (Fig. 1). A threshold is applied to the partitions, which divides the partitions into a positive and a negative population. The ratio between the number of positive and negative partitions is used to calculate the absolute number of target molecules, corrected for the chance that partitions are shared by multiple target molecules by the Poisson distribution law [2]. The first steps towards digital PCR were taken 30 years ago when the concept of limiting dilution and Poisson distribution were applied to detect rare targets [3–5]. In the field of HIV research, Simmonds et al. [6] combined PCR with limiting dilution to quantify the proviruses in HIV-infected cells. The term ‘digital PCR’ was introduced by Vogelstein in [7] to identify specific mutated sequences in a minor fraction of a cell population. Nowadays digital PCR is a widely accepted quantification tool and applied in many fields.

## Digital PCR platforms

The key principle of digital PCR is the distribution of a sample among multiple partitions. Originally, partitions were created by manually distributing a sample over a number of wells [7]. Nowadays, manual partitioning is applied in case of complex protocols with a nested approach that cannot be adopted to an automated platform, such as the digital PCR described as a manual repetitive sampling protocol that is used to measure integrated HIV DNA [8, 9]. However, manually generating multiple partitions is very time-consuming and laborious. The past decade automated systems have emerged and different technologies and methods are being explored by various companies for digitizing PCR (for an overview, see Table 1). Currently available digital platforms differ in number of partitions, method of generating partitions or required specialized equipment. Partitions can be generated in a pre-manufactured array: BioMark™ HD System (Fluidigm) provides a wide range of specialized digital integrated fluidic circuits (IFCs) arrays where the sample is dispensed in a well and distributed over multiple individual reaction chambers. QuantStudio 3D (Life Technologies/Applied Biosystems™) employs a silicon chip that consists of a single array of individual reaction wells onto which the sample is dispensed. CONSTELLATION^®^ Digital PCR System (Formulatrix) utilizes a microplate where connecting channels are isolated into individual microfluidic chambers by a seal-compressing roller. In contrast to these array-based approaches, other digital PCR platforms such as the QX200™ Droplet Digital™ PCR (ddPCR) and RainDrop *plus*™ Digital PCR system (RainDance™ technologies) use water-in-oil emulsion chemistry to create partitions. The aqueous phase consisting of primers, probe and supermix, sample, and a mineral oil is loaded into a specifically designed holder. The droplet generator uses microfluidics to create a pressure that draws the aqueous and oil phase into the output channel, forming the droplets in the process. Each droplet is read one by one in a specialized droplet reader. Finally, Naica system from Stilla combines both the array and emulsion approaches. In this system, a sample runs through the channels of a chip and droplets are created inside the chip.

**Table 1:** Characteristics of different digital PCR platforms. Information is extracted from companies’ website unless cited otherwise

	Biomark	QuantStudio™ 3D	Naica	RainDrop plus™	QX200™ Droplet Digital™ PCR	CONSTELLATION^®^ DPCR
Company	Fluidigm™	Applied Biosystems/Life technologies™	Stilla Technologies	RainDance™ Technologies	Bio-Rad	Formulatrix
Type	Integrated fluidic circuits (IFCS) arrays	Chip	Crystal droplets in an array	Picosized droplets	Nanosized droplets	Microfluidic chambers
Detection mode	Real-time and end-point	End-point	End-point	End-point	End-point	End-point
Chip/partitions consumables	qdPCR 37 K IFC, 48.770 and 12.765 Digital Array™ IFC	QuantStudio™ 3D Digital PCR Chip (v1/v2)	Sapphire chip	RainDance Source Chip	Microfluidic cartridge	CONSTELLATION^®^ Digital PCR System microplate
Number of samples (max)						
Loading	48/12 samples per array	1 sample per chip	4 samples per chip	8 samples	96 samples	24/96 samples
Cycling	48/12 samples	24 samples	3 chips or 14 samples	96 samples	96 samples	24/96 samples
Reading	48/12 samples	1 sample per chip	3 chips or 14 samples	8 samples	96 samples	24/96 samples
Input volume per sample	4 µL/8 µL	14.5 µL	25 µL	25–50 µL	20 µL	10 µL
Reactions per sample	770/765	20,000	25,000-30,000	5–10 million	20,000	36,000/8000
Reaction volume per partition	0.85 nL/6 nL	0.755 nL (v2 chip) or 0.809 nL (v1 chip)	0.43 nL	5 pL	0.868 nL [10]	NA
Specialized equipment	IFC controller Biomark (thermal cycler and reader)	ProFlex™ 2 × Flat PCR System QuantStudio™ 3D Digital PCR Instrument	Naica Geode (thermal cycler) Naica Prism3 (reader)	RainDance Source/ThunderBolts™ System RainDrop^®^ Sense Operator	QX200™ Droplet Generator QX200™ Droplet Digital™ PCR System	CONSTELLATION^®^ Digital PCR System
Software	Digital PCR Analysis	QuantStudio™ 3D AnalysisSuite™	Crystal Miner	RainDrop Analyst II™	QuantaSoft™	CONSTELLATION^®^
Detection	Detection of up to 3 fluorescent dyes per assay	2 detection channels (FAM/VIC)	3 color detection (FAM/VIC/Cy5)	2 detection channels (FAM/VIC)	2 detection channels (FAM/VIC)	5 probe wavelengths per sample

## Challenges and benefits of droplet digital PCR

**Fig. 2:**
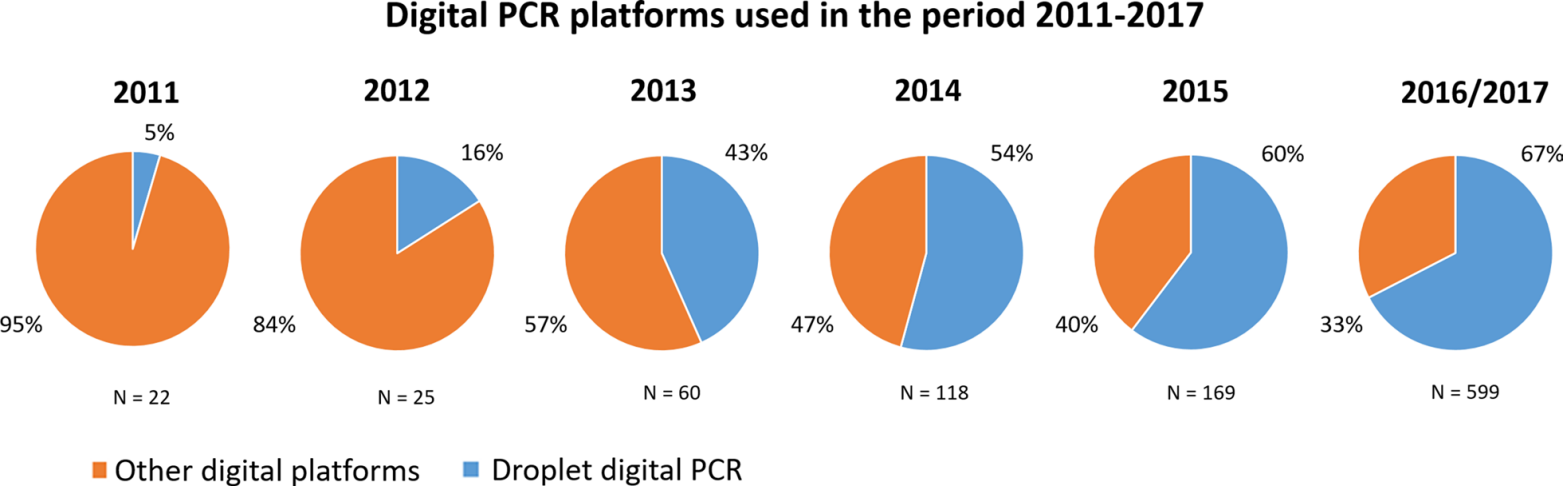
The use of droplet digital PCR during the period 2011–2017, reported as percentage of total number of digital PCR articles cited in PubMed (search terms: “digital PCR” or dPCR, droplet digital PCR” or ddPCR)

There are multiple digital PCR platforms, but over the past years, the QX200 has steadily become the most widely used digital PCR platform across all research fields (Fig. 2). Therefore, in this review we will focus on the QX200 ddPCR from Bio-Rad to discuss challenges and benefits of digital PCR. It should however be noted that challenges with threshold determination and false-positives are not exclusively observed with the ddPCR from Bio-Rad but seem to be related to other digital platforms as well [11–15].

### Threshold determination

In ddPCR generated droplets are identified as positive or negative based on a threshold at a certain fluorescence level and this ratio is used to calculate target abundance using Poisson-statistics. Therefore, determining a correct threshold is crucial for reliable quantification (Fig. 1). Defining a threshold is complicated by droplets with an intermediate fluorescence, termed as rain, which are puzzling to assign to the either positive or negative population. For the frequently used Bio-Rad ddPCR system, the QuantaSoft software offers an undisclosed method for automated threshold assignment and manual threshold setting by the end-user. The automated analysis often assigns thresholds so strict that a cloud of droplets is appointed positive that based on their low fluorescence is expected to be negative [16]. Alternatively, user-defined thresholds may be applied but these are generally not advised as they impair an unbiased interpretation of digital PCR data. Threshold setting can be challenging since the separation between positive and negative droplets may depend on many factors, such as the quality and quantity of the input sample, melting temperature and length of primers and probe, mismatches between the assay and target sequences, time between droplet generation and readout, pipetting precision, type of fluorescent reporter and type of quencher. Several algorithms have been developed by end-users that aim to offer more data-driven approaches to set thresholds. First, clustering methods were developed by Strain et al. and Jones et al. based on k-nearest neighbor-joining [17, 18]. The method of Strain et al. defines the median and variance of the negative and positive clouds to assess the statistical likelihood that outliers should be included in either cloud (*p* < 0.1). Jones et al. developed “definetherain” that uses negative and positive control samples to identify the two clouds. Subsequently, the mean fluorescence minus or plus three times its standard deviation is used as thresholds that are applied to the samples. Both these clustering methods calculate a threshold for each cloud of droplets and exclude intermediate fluorescent droplets from further analysis. In contrast, Dreo et al. proposed a single threshold determination method since droplets with intermediate fluorescence intensity can hold true positive droplets [19, 20]. This global manual threshold is defined as the mean fluorescence signal in the NTCs (no template controls) plus a number of standard deviations until one positive droplet remains in the NTCs [19]. These described methods assume a normal (binomial) distribution of the negative and positive clouds and do not account for shifts in baseline fluorescence between droplet populations of different samples. However, distribution fitting experiments and normality testing shows that droplet clouds do not follow a normal distribution and cannot be described by a single family of distributions. Furthermore, baseline fluorescence of the negative cloud has been shown to vary between samples and influence quantification [16]. Therefore, an alternative thresholding method was devised by Trypsteen et al. [16] that assigns a threshold regardless of the many factors that may affect the intensity and distribution of droplet fluorescence. This method, ddpcRquant, feeds data from negative controls to a generalized extreme value model and applies this threshold to the samples. The algorithm does not make assumptions of the underlying distribution of the droplet populations and accounts for baseline shifts. Alternatively, Lievens et al. [20] determine the threshold based on the shape of the fluorescence density peaks but to account for the possibility that clouds are not normally distributed set the threshold above the uppermost limit of the negative cloud. Recently, a novel method, “Umbrella”, was published that does not apply hard thresholding, but applies a model-based clustering and takes partition-specific classification probabilities into account to produce a final quantification result [21]. Threshold setting remains a challenging but crucial task. It is difficult to establish whether or not intermediate droplets represent true targets that should be used for analysis, since the current generation of ddPCR is not fitted with a fluorescence intensity sorter to allow for target confirmation by for example sequencing. Recent evidence however suggests that intermediate droplets should be considered to contain target molecules, as decreased amplification efficiency may arise from a suboptimal annealing temperature [22] or mismatches between the assay and the target sequence [16]. Furthermore, several studies that investigated ddPCR sensitivity have used a user-defined threshold that allocates rain to the positive fraction of droplets, and doing so have found results that are on par with the input reference and qPCR results [11, 20, 23, 24].

### False-positives

Regardless of the method that is used to assign a threshold, currently available digital PCR platforms including the QX200 suffer from the observation of false-positive partitions and therefore false-positive results [11, 16, 18, 23–25]. One out of three wells of negative controls with no template had 2 or 3 positive droplets (0.16–0.22 copies/reaction) for HIV-1 RNA assay described by Kiselinova et al. [23]. These droplets had a similar fluorescence level as positive droplets in patients samples. The origin of these errors remains unclear and various hypotheses have been proposed. False-positive droplets can arise from contaminations or disturbed droplets that merge together, their joint fluorescence leading to a droplet with a higher baseline fluorescence that is miscalled as positive.

False-positive droplets can pose a threat to reliable HIV DNA quantification in settings with low HIV DNA concentrations such as mother-to-child transmission, early treatment initiation and allogeneic stem cell transplantation (alloSCT). AlloSCT is currently the only known approach by which the HIV reservoir can be drastically reduced. Following a successful stem cell transplantation, patients are kept on ART and are monitored for HIV DNA levels, but reliable ascertainment of remainder HIV DNA is a challenge, especially when the interpretation of true-positive droplets is obscured by false-positive ones. Same holds true for ART-treated children, which may have initiated ART early after birth based on the HIV-status of their mother whereas uncertainty may exist if the infection was transmitted from mother to child. In these seronegative children, HIV DNA is the only proof of HIV infection and therefore the only justification for treatment with ART. However, confirmation of the presence of HIV DNA is challenging since patients who initiated ART early after infection are known to have small reservoirs and sample volumes are restricted in case of young children, which reduces the statistical power to assess the presence of HIV DNA. Therefore, false-positives can unrightfully lead to confirmation of HIV infection and continuation of ART and it is not advised to use digital PCR if the question is to discriminate between presence or absence of HIV DNA [11]. Since only a minor fraction of all potential CD4 positive target cells carry HIV DNA, a large number of cells need to be tested in order to be able to reliably quantify HIV DNA concentrations. High concentrations of total DNA however affect the viscosity of the aqueous phase and complicate the formation of droplets. The amount of DNA that can be loaded into a single reaction is therefore restricted [18, 26]. Researchers who aim to report an HIV DNA concentration in a million CD4-cells are required to split the target DNA among a number of reactions, thereby increasing the risk of detecting false-positive droplets and influencing final HIV DNA concentration outcome. This effect is even greater when samples are used in which HIV DNA is even less abundant, such as PBMCs, whole blood, dried blood spots or tissue biopsies.

### Advantages

Apart from the issue of false-positives, digital PCR has shown to be equal or superior to qPCR in several aspects. One major advantage is that digital PCR produces direct absolute quantification. The absolute quantification results produced by digital PCR eliminate the need for a standard curve in case of DNA quantifications and comparisons of RNA quantifications. Of note, RNA quantification represents cDNA molecules and should therefore be corrected for cDNA synthesis efficiency [27]. Accurate quantification by qPCR is based on the quality of the standard curve: instability of the standard curve can lead to inaccurate HIV DNA quantification [28]. Additionally, Cq values in qPCR that arise from the standard and the samples are based on amplification efficiencies, and several factors may confound their correct interpretation. Amplification efficiency may be affected by inhibitors, amount of total DNA that is loaded as well as variation between the primer/probe and the patient’s viral sequence, and these factors may unrightfully elevate the Cq values. In qPCR, such mismatches would increase the Cq and in turn present a target abundance that is lower than the actual input. In ddPCR however, a reduced amplification efficiency leads to less fluorescence at end-point. As long as end-point fluorescence remains above the assigned threshold and the ratio between positive and negative fraction of droplets is unaltered, mismatches between assay and target are allowed as they do not affect the quantification outcome [16, 29, 30]. Tolerance to target sequence variation is especially crucial for HIV quantification as a higher chance of mismatches with the primer–probe set is to be expected due to high heterogeneity of the virus [31]. Besides the robustness of ddPCR with respect to inhibition and reduced amplification efficiency, a higher precision and reproducibility was observed for ddPCR in comparison to qPCR [18, 32]. This is especially crucial in HIV cure efforts where the aim is to detect potential effects of the interventions on the HIV reservoir. However, it is important to note here that contradicting findings have been published that observed a higher sensitivity of the qPCR platform [23, 33]. In duplex digital PCR experiments on linked targets, a minority of partitions was observed in which only one out of two assays demonstrated amplification [34]. It remains however unclear whether this observation is artificial due to DNA shearing and physical separation of supposed linked targets, or genuine failure to amplify due to assay-specific inhibitors, DNA degradation or tertiary structures. Furthermore, in case of genuine failure to amplify it is currently unclear whether this potential mode of target underestimation pertains to digital PCR alone or if similar mechanisms are at play in case of (q)PCR.

## Applicability and future perspectives

HIV reservoir measurement by digital PCR has been used to measure the effects of early treatment initiation [35–38], therapeutic vaccination [39–41], allogenic stem cell transplantation [42], structured treatment interruptions [40, 43], immunization by broadly neutralizing antibodies [44], latency reversing agents (LRA’s) [41, 45–49], and other novel therapeutic agents [50–52]. The concept of digital PCR is well-established but automated platforms and implementations in HIV quantification are relatively recent and the field is looking forward to future advancements. Where some platforms limit the number of specialized devices needed (CONSTELLATION^®^ Digital PCR System microplate from Formulatrix), other companies are working on a multiplex system up to 6 colors (Naica system from Stilla) or enable the analysis of multiple samples in a single run (QX200 from Bio-Rad). The combination of these features combined in a single device with a high-throughput workflow and elaborate multiplex system is desired. In addition, data analysis and threshold setting should be further developed in order to keep up with advances in multiplexing. Considering the observed false-positive partitions in current digital PCR platforms, quality control of the partitions is crucial. Naica system from Stilla currently allows visual inspection of the size and geometry of a single crystal droplet and the exclusion of those that are aberrant. The QX200 digital PCR platform may benefit from an integrated fluorescence sorter for post-PCR analysis of droplets. Such a feature would improve our understanding of the nature of suspected false-positive droplets by allowing post-PCR sequencing to verify if fluorescence is the cause of PCR or rather through the acquisition of fluorescent dust or debris. In addition, post-PCR sorting of single-cell droplets may improve our understanding of the dynamics involved in latency [47]. Yucha et al. demonstrated that the QX200 cartridge can be used to create single-cell-droplets, after which HIV RNA was quantified using standard digital PCR protocol. Using a blunt needle, they manually selected positive droplets for post-PCR sequencing of HIV ENV and human CCR5, and future experiments may even investigate HIV integration site or viral protein production. This holistic approach to HIV latency research holds great promise, yet requires specialized equipment and trained personnel and would therefore benefit from a fluorescence sorter that is integrated into the QX200 reader. Although digital PCR allows the precise quantification of HIV DNA and RNA in patients, it does not enable researchers to gain information about the replication-competence of the reservoir. Whereas the cell culture based viral outgrowth assay is an underestimation of the true viral reservoir, HIV measured by PCR is an overestimation because it counts the non-replication competent viruses as well [53]. Multiplexed ddPCR may however improve our understanding of the gap between viral outgrowth and PCR-based assays. Anderson et al. [54] used a multiplexed ddPCR to observe an increase of the LTR:gag ratio during time on treatment, which can be explained by elimination of replication-competent viruses or clonal expansion of non-replication competent viruses. Additionally, multiplexed ddPCR could aid in determining the number of times an HIV sequence has been clonally expanded. Clonal expansion and its specific HIV integration site is an international focus point since it is linked to persistence of HIV-infected cells [55]. However, integration site analysis is laborious and expensive but designing a multiplex ddPCR that targets HIV and the human sequence adjacent to HIV, clonal expansion of that specific HIV sequence can be calculated based on the increase of double-positive droplets relative to expected number based on chance [56]. In summary, digital PCR has proven to be a valuable new technology and with additional improvements in prospect it is likely to mature into an indispensable tool in future HIV research.

## Abbreviations

ART: antiretroviral therapy
ddPCR: droplet digital PCR
qPCR: real-time PCR
LRA: latency reversing agent
